# WHEN DISASTER STRIKES: ADDRESSING THE UNIQUE NEEDS OLDER ADULTS FACE IN EMERGENCY SITUATIONS

**DOI:** 10.1093/geroni/igad104.0418

**Published:** 2023-12-21

**Authors:** Amaanali Fazal, Kahir Lalji, Barbara McMillan

**Affiliations:** United Way British Columbia, Burnaby, British Columbia, Canada; United Way British Columbia, Burnaby, British Columbia, Canada; United Way British Columbia, Burnaby, British Columbia, Canada

## Abstract

Older adults are more at risk for harm in emergency situations (e.g., pandemics, natural disasters, extreme weather events) and often require more assistance in response and recovery efforts. The COVID-19 pandemic and the increase in climate related emergencies has emphasized the critical need to prepare and respond more effectively to the unique needs of older adults in emergency situations. There is much that community-based seniors serving (CBSS) organizations, emergency management professionals, and local governments can do to develop and implement policies, programs, and practices to effectively support the needs of older adults in emergency preparedness and response. In British Columbia, the COVID-19 pandemic and recent extreme weather events (i.e., forest fires, flooding, and heat waves) have prompted the development of an intentional network strategy for emergency preparedness and response. This innovative approach, which is coordinated by United Way British Columbia, has allowed for increased communication, coordination, collaboration, peer learning, and knowledge translation among CBSS agencies, as well as partner organizations, allied professionals, and other stakeholders. This presentation describes our comprehensive approach to supporting communities to understand and address the specific needs of older adults in emergency planning and management, including: 1) community grants and capacity building; 2) tools and resource development; 3) policies and rights-based strategies; and 4) fostering collaboration between CBSS agencies, emergency management professionals, and all levels of government. Examples are also provided illustrating how these approaches have been utilized in British Columbia to respond to the COVID-19 pandemic and severe weather events.

